# Management of Cardiac Toxicity Induced by Chemotherapy

**DOI:** 10.3390/jcm9092885

**Published:** 2020-09-07

**Authors:** Dario Trapani, Paola Zagami, Eleonora Nicolò, Gabriella Pravettoni, Giuseppe Curigliano

**Affiliations:** 1 European Institute of Oncology, IRCCS, 20141 Milan, Italy; dario.trapani@ieo.it (D.T.); paola.zagami@ieo.it (P.Z.); eleonora.nicolo@ieo.it (E.N.); Gabriella.Pravettoni@ieo.it (G.P.); 2 Department of Oncology and Hematology (DIPO), University of Milan, 20122 Milan, Italy

**Keywords:** cardiotoxicity, chemotherapy, cardio-oncology, treatment-related adverse events, pharmacotherapy

## Abstract

Cardiotoxicity encompasses a spectrum of adverse cardiological effects experienced by cancer patients during and after receiving antineoplastic treatments. The intersection of cancer care with the management of the multiple comorbid non-communicable diseases carried by patients or related to cancer treatments motivates the need for an integrated and multidisciplinary approach to therapeutic clinical decision-making. This present review aimed to provide a perspective and an update of the current pharmacotherapy approaches for the prevention and management of cardiotoxicity from antiblastic chemotherapy; as such, it addresses myocardial, vascular, and arrhythmic disorders associated to chemotherapy, by navigating the current knowledge and clinical indications in support of the medical interventions. Clinical scenarios of pharmacological interventions take place with patients receiving anthracycline and, by extrapolation, other agents with cardiotoxic potentials and non-chemotherapy agents, including various small molecules and immunotherapy agents. Analysis of these scenarios aims to provide practical evidence-based guidance for the management of drug-induced cardiac dysfunctions. The possible role of new biomarkers for the early recognition of cardiotoxicity is mentioned across the clinical studies, with reference to the pharmacological biomarker-driven interventions delivered. To best inform survivorship care, the management and context of cardio-oncology services are discussed within the broader network of providers and settings of care.

## 1. Introduction

**Figure 1:**
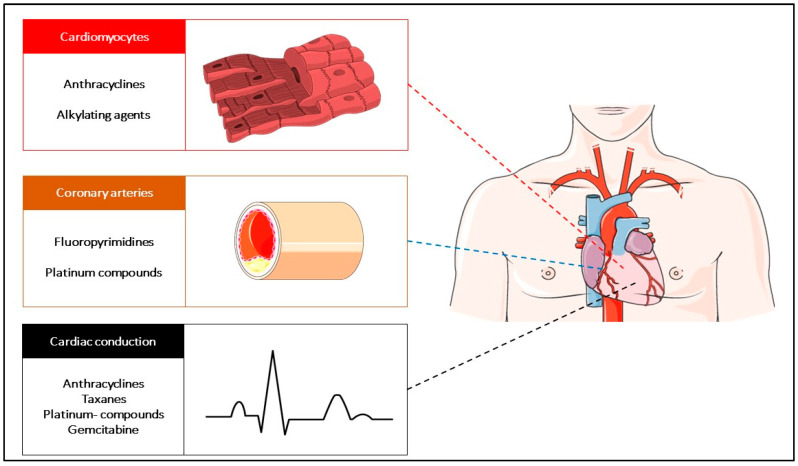
The spectrum of cardiac toxicity and the most common related causative antineoplastic agents.

The improvement of early diagnosis and timely cancer treatments has resulted in an improvement of the cancer survival rate in the last decade, with a growing population of cancer survivors. Cancer survivors can carry a multitude of treatment-related comorbidities, including cardiovascular toxicities [1]. As a consequence of treatments, cardiac and vascular pathological conditions can arise de novo from identifiable causal therapeutic agents or emerge for clinical consideration as a worsening of a previously known comorbidity. The presence of comorbidities has a prominent prognostic significance for cancer patients, independently affecting the survival outcomes [2,3]. However, the quality of data used to guide the clinical decisions regarding the appropriate clinical strategies of prevention and control of the cardiovascular sequelae in cancer survivors is still scarce and is widely based on experts’ consensus. This motivates the urgent need for research in cardio-oncology. This present review aims to discuss key points for the management of patients treated with chemotherapy agents and experiencing one or more cardiovascular treatment-related toxicities at some point during the care process. The scheme used in the present work is based on the following tripartite approach for cardiovascular disease description and reporting: alterations of the contractile myocardium, insufficiency of the vascular supply, and electric heart disorders. The toxicity caused by anti-vascular and targeted agents or immune agents are outside the primary scope of this review and will only be briefly mentioned, addressing the analogies and divergencies with chemotherapy (Figure 1).

## 2. The Setting for the Management of Chemotherapy-Related Cardiotoxicity

Cancer health providers are generally aware of the acute onset of the cardiovascular toxicities related to chemotherapeutics or selected monoclonal antibodies and small molecules, and to some extent, of the myocardial effects from immunotherapy agents. However, the management of long- term sequelae is often transferred to the primary health setting (PHS) [4,5,6,7,8]. A multitude of cardiovascular sequelae can emerge as clinically relevant entities during or beyond the temporal window of a standard follow-up, generally between 5 and 10 years after the cancer treatment completion [9]. The current international guidelines for the estimation of cardiovascular risk generally do not account for the history of cancer and the receipt of previous treatments, such as chemotherapies (i.e., type and exposure) and radiation therapy (i.e., volume and dose) [10,11]. Therefore, the development of an adjustable tool that is ready for clinical utilization to better tailor the needs of cancer patients for the screening and prevention of the cardiovascular spectrum of diseases, framed in a comprehensive approach, is urgently required [12]. Furthermore, the identification of patients at higher risk to experience cardiac toxicities or develop long term sequelae is a healthcare priority, which requires an approach that involves the continuum of care and a pluralistic dialogue between clinicians from the referring PHS to specialistic care [13].

## 3. Principles of Treatment of the Chemotherapy-Induced Cardiotoxicity

### 3.1. Prevention of Cardiovascular Damage

Patients with no baseline cardiovascular impairment, no increased risk for cardiovascular events, and a conserved left ventricular ejection fraction (LVEF) can benefit from primary preventive pharmacotherapeutic interventions [14]. Primary prevention with antidotes against cardiotoxic agents has been proposed in the past, particularly when patients are exposed to high doses of cardiotoxic agents. Anthracyclines are the most prominent cancer treatment agents that cause heart damage, which is expressed mainly, but not exclusively, as left ventricular dysfunction, especially in the symptomatic stage of the damage [15]. The benefit of anthracyclines in some tumors, for example, lymphoma, leukemia, and breast cancer, has been thoroughly established but the onset of cardiac toxicity or cardiological contraindications for anthracyclines can result in a poorer oncological outcome, which emphasizes the need to maintain the treatment intensity while ensuring the safest delivery for patients [16]. The most common pattern of toxicity from anthracyclines is cumulative, dose-dependent, and not reversible [17]. Studies on a large mix of cohorts of cancer patients have reported cardiotoxicity occurring in 1 patient out of every 10 treated with chemotherapy, mainly during or just after the completion of the treatments [18]. 

#### 3.1.1. An Antidote to Reduce the Direct Damage of Cytotoxic Drugs: The Dexrazoxane Paradigm

Dexrazoxane is a derivative of the chelating agent ethylenediaminetetraacetic acid, which is an iron chelator that can reduce the generation of oxygen radical species [19]. Radical stress mediated by superoxide species is considered the principal etiopathogenetic mechanism of cardiac damage in the presence of anthracycline. Though it is still unclear how anthracyclines damage cardiomyocytes, the prevention of the formation of anthracycline complexes with metal ions seems to dampen the release of harmful free radicals, reducing cardiac damage [19]. The use of dexrazoxane as a cardioprotective agent has been included in the clinical protocols since the 1980s. However, in 2011, the European Medicines Agency issued a re-analysis of the safety profile of this agent, raising possible concerns regarding its association with increased risks of infection, myelosuppression, and secondary malignancies, mainly hematologic, with special relevance to pediatric use, contraindicating the drug for children and adolescents due to insufficient evidence of safe activity [20]. In adults, the role of dexrazoxane has been systematically documented in a Cochrane Group review, pooling the data from ten clinical randomized trials on 1619 patients [21]. The meta-analysis showed a statistically significant benefit in favor of dexrazoxane relative to the occurrence of heart failure (relative risk: 0.29; 95% CI: 0.20–0.41) and confirmed the oncological safety of this drug. No evidence for a difference in response rate, survival, or occurrence of secondary malignancies between the dexrazoxane and control groups was demonstrated [21]. When used as part of the chemotherapy of cancer patients, dexrazoxane has been shown to provide acute cardioprotection, based on surrogate markers of cardiac damage, such as troponin-T levels and echocardiographic measurements. However, no long-term data proving a reduction of the risk for symptomatic heart failure in children receiving high-dose anthracyclines has been reported [22]. In adults, the use of dexrazoxane is approved for restricted clinical indications, namely, in patients for which the cumulative dose of anthracycline is anticipated to be higher than 300 mg/m^2^ of doxorubicin (or 540 mg/m^2^ of epirubicin) [23]. In particular, in Europe, the use is limited to metastatic breast cancer patients when the cumulative dose exceeds 300 mg/m^2^ of doxorubicin but anthracyclines are still used [24]. 

#### 3.1.2. Targeting the Key Pathogenetic Mechanisms of Chemotherapy-Induced Cardiotoxicity

The activation of the renin–angiotensin–aldosterone hormone system (RAAS) has been suggested as playing a central role in the physiopathology of the anthracycline-induced chain of cardiotoxic events. RAAS is a finely controlled biological system for the maintenance of ions, fluid balance, and blood pressure [25]. The system is made up of protein fractions in the plasma: renin, an enzyme secreted by specialized juxtaglomerular kidney cells in response to hemodynamic and neuro-endocrine stimulations; angiotensinogen, produced by the liver and converted by renin into angiotensin I; angiotensin-converting enzyme (ACE), an enzyme that cleaves angiotensin I into angiotensin II, which exerts biological activities via receptor type I and type II, and regulates the adrenal incretion of aldosterone. At the level of cardiomyocytes, RAAS has a role in the cellular stress response that is involved in the pathogenesis of pathological heart remodeling in patients carrying chronic cardiotoxic sequelae [26]. Therefore, the use of angiotensin-converting enzyme inhibitors (ACE-I) or angiotensin receptor blocker (ARBs or “sartans”) represents a cornerstone in the prevention and treatment of cardiovascular sequelae of a multitude of risk-increasing conditions, including chemotherapy [26].

The blockade of the RAAS using the endocrine effector aldosterone has been identified as an appealing strategy in this context. Spironolactone is a steroid with anti-aldosterone properties, which is used in clinical settings for multiple indications of primary or compensatory hyper-aldosteronism, including heart failure. A Turkish clinical trial tested the cardioprotective value of spironolactone, enrolling breast cancer patients (5–15% metastatic patients) receiving adriamycin or epirubicin-containing chemotherapy regimens [27]. Patients were monitored with two-dimensional trans-thoracic heart ultrasonography for the estimation and monitoring of the LVEF. The investigators observed that the LVEF decreased from 67.0 ±  6.1% to 65.7  ±  7.4% (*p*  =  0.094) in the spironolactone group and from 67.7  ±  6.3% to 53.6  ±  6.8% (*p*  <  0.001) in no spironolactone group, reporting an attenuation of the LVEF suppression from the baseline when the cardioprotective drug was added. An exploratory analysis found a correlation between the LVEF and the cumulative dose of both the doxorubicin and epirubicin anthracyclines (*r* = 0.66–0.72), hypothesizing a cumulative dose effect. 

The manipulation of RAAS has also been achieved using receptor blockers of the peptide hormones. The PRADA (PRevention of cArdiac Dysfunction during Adjuvant breast cancer therapy) study on the prevention of LVEF dysfunction enrolled patients with breast cancer who were treated with adjuvant chemotherapy-containing anthracyclines, with or without trastuzumab, and locoregional radiation therapy (*n* = 120 patients). These patients received the ARB candesartan cilexetil, the beta 1 selective adrenergic blocker (bB) metoprolol succinate, or a combination of them [28]. PRADA was designed as a 2 × 2 factorial study (beta-blocker vs. ARB or cardioprotective therapy vs. no protective therapy), with the aim to measure the change in LVEF from baseline to the completion of the adjuvant anticancer therapy, as determined using cardiac magnetic resonance (MRI). The study population presented a low percentage of co-morbid conditions or cardiac risk factors at baseline. For patients receiving candesartan, the drug attenuated the LVEF decline by 1.8% compared to the placebo, for patients both with and without baseline hypertension. The incorporation of metoprolol was not associated with a significant adjunctive protective effect in terms of an LVEF change (*p* = 0.77). Overall, the combination of the two drugs failed to show a synergistic effect. The OVERCOME (preventiOn of left Ventricular dysfunction with Enalapril and caRvedilol in patients submitted to intensive ChemOtherapy for the treatment of Malignant hEmopathies) trial assessed the role of the ACE-I enalapril plus the beta 1, beta 2, and alpha 1 adrenergic receptor-blocker carvedilol in patients with acute leukemia or generic blood malignancies, such that they were eligible for autologous hematopoietic stem cell transplantation, and with the absence of baseline left ventricular dysfunctions [29]. The study was conducted in a single institution, on a Catalonian cohort of patients, and was designed to compare the absolute change from baseline LVEF with or without the pharmacological intervention (*n* = 90 patients). In the first semester of treatment, significant reductions in the echocardiography- and MRI-estimated LVEF was observed between the control versus the experimental group of 3.1% and 3.4%, respectively, which was essentially driven by patients with acute leukemia. More interestingly, an explorative analysis showed an absolute difference in cardiovascular death or clinically significant heart failure of −15.3% with enalapril and carvedilol. However, such analysis was only hypothesis-generating, as it was not preplanned. To better understand the role of bBs in the setting of the prevention of organ dysfunction in patients receiving cardiotoxic drugs, an adequately powered study has subsequently been designed with the use of carvedilol, namely, the CECCY (Carvedilol for prEvention of Chemotherapy-related CardiotoxicitY) trial (*n* = 200). [30] This trial enrolled breast cancer patients whose treatment was initiated with anthracyclines [30]. The study was designed to test the alternative hypothesis that the use of carvedilol would prevent systolic dysfunction (defined as a ≥10% reduction in the LVEF) at 6 months. In the short follow-up term established per trial, there was no significant difference in the primary outcome in patients receiving or not receiving the preventive intervention, meaning the null hypothesis could not be rejected. The investigators reported a higher likelihood of troponin I pathological increase over time in the non-interventional group, suggesting a benefit regarding the marker of myocardial necrosis. Furthermore, they noted a lower incidence of diastolic dysfunction in the interventional group. Taken together, the results warrant a longer follow-up, as the troponin increase and diastolic dysfunction have been proposed as early markers of cardiac damage that are capable of anticipating a subsequent occurrence of heart failure [31] (Table 1). 

### 3.2. The Management of Cardiotoxicity Occurring during the Cancer Treatments: The Clinical Scenarios

#### 3.2.1. Anthracycline-Related Damage: Targeting the Direct Cardiomyocytes Toxic Agents

The spectrum of cardiotoxicity during cancer treatments can be variable and protean. While the onset of clinically critical adverse events can prompt the discontinuation of oncological therapies, the decision to discontinue permanently potentially life-saving therapeutics can still be challenging. Where clinically appropriate, the cardiological schedule of monitoring should be adjusted for the risk of toxicity, based on a baseline risk estimation and the known potentiality of cardiotoxicity, which dictates the intensity of both the types of surveillance strategies and the follow-up schedules [24]. Several clinical scenarios can be defined to provide pharmacotherapy for preventing severe sequelae and treat specific events. We pooled some scenarios of common clinical presentations. 

##### Clinical Scenario 1: Asymptomatic Patients Receiving Anthracycline

This scenario involves those patients receiving chemotherapy and experiencing an LVEF decrease of ≥10% from baseline to below 50% and for all experiencing a decline of ≥40% but <50%. In these patients, the (at least) temporary interruption of the treatment is mandated, and cardiologic therapy should be considered to rescue the LVEF function before resuming the cancer treatments [24]. When no intervention is provided, the probability of having a spontaneous rescue of the left ventricular systolic function is poor (less than 10%). One prospective study on a Danish cohort of advanced breast cancer patients analyzed the patterns of the decline of LVEF via serial measurements of the left ventricular systolic function [33]. The study showed no improvement in the LVEF in patients experiencing a deterioration of the cardiac function during 3 months of digitalo-diuretic therapy. However, ACE-I therapy was able to rescue the LVEF in all the patients and stabilize the heart function in the long term. When ACE-I was added to the treatment, no hypotension was observed, suggesting that a low dose of ACE-I can be safely administered in patients with normal baseline arterial pressure, in the absence of major contraindications. Another study designed for cancer survivors assessed the value of the ACE-I enalapril at a low dose (0.098 mg/kg/die) against a placebo in a double-blind, controlled clinical trial [34]. The target population consisted of patients aged 8 years and older who developed cancer before the age of 20 years and were previously treated with anthracyclines for 2 or more years from the completion of the cancer treatment. The authors demonstrated a significant rate of change in the left ventricular end-systolic wall stress (LVESWS). LVESWS is an index of afterload, which is proposed as an early marker of chemo-induced remodeling and heart failure. Though an improvement of the LVESWS was observed, no change in the systolic function was detected in the exploratory analysis, therefore providing inconclusive results for this population. To date, the administering of protective agents to all-comer childhood cancer survivors with preclinical modifications of the heart structure or function does not seem to be supported and all the decisions should be based on clinical findings and based on survivorship guidelines. The single-arm Italian study from Cardinale et al. assessed the combination strategy of enalapril (2.5 to 20 mg) and, when indicated and tolerated, carvedilol (6.25 to 50 mg) for patients experiencing an LVEF decline below 45% due to the exposure to anthracyclines [35]. The use of ACE-I plus bB resulted in the complete reversal of the LVEF decline in nearly half of the population studied, all of which occurred within the first 6 months of the cardiological treatment. However, for 45% of the patients, no response was observed. Interestingly, patients with the total reversion of the systolic dysfunction experienced the best clinical course, with a low rate of cumulative cardiac events, including death. According to these data, the current guidelines state that when experiencing an asymptomatic LVEF drop, the introduction of ACE-I can be recommended. So far, no trial has demonstrated the benefit of including bB or a specific role as a single agent; for ARBs, their value should be calculated by extrapolating from the data of ACE-I, as no trial has been specifically designed with these agents. The current indication is to consider ARB when ACE-I are contraindicated or poorly tolerated, as it is suggested in clinical practice. 

The use of cardioprotective agents has been proposed based on biomarkers of early damage of the heart that are capable of reliably predicting a significant LVEF decline and heart failure. The decrease of the average global longitudinal strain from baseline assessed using 2D heart ultrasonography, as well as the rise of cardiac troponin, have been proposed as biomarkers of damage. In some cases, considerations of cardio-protectants can be provided based on surrogate biomarkers, especially when the pathological changes persist after repeated measurements and in frail or multi-comorbid patients [24]. Currently, the ICOS-ONE study (Anthracycline-induced cardiotoxicity: a multicenter randomised trial comparing two strategies for guiding prevention with enalapril: The International CardioOncology Society-one trial) investigated the opportunity to develop a troponin-triggered ACE-I cardioprotective treatment versus ACE-I to all the patients receiving anthracyclines [36,37]. The study failed to show a benefit of the primary prophylaxis with ACE-I, as no differences across the arms were reported in terms of a cardiac troponin rise. The authors reported a possible role of ACE-I in patients experiencing pre-clinical heart damage, e.g., with a rise of troponin. Nevertheless, a minor elevation in troponin levels should be considered normal, especially when no change in the LVEF is observed; such a situation usually warrants only clinical monitoring, as it is self-limiting in nature. 

For patients pre-exposed to anthracyclines and experiencing asymptomatic sequelae, the international clinical guidelines suggest the inclusion of liposomal formulations to reduce the cardiac toxicity [24]. Though based on low-quality evidence, liposomal doxorubicin has often been used in clinical practice for patients with an anthracycline-related suppression of the LVEF, though otherwise asymptomatic, in which the benefit of anthracyclines is deemed to outweigh the risks. Liposomal formulations of doxorubicin have been identified as one of the key strategies for preventing the occurrence of treatment-related heart failure in patients at higher risk due to their need for a safer cardiological profile [24]. In fact, the use of liposomal delivery is thought to have the same value as dexrazoxane in terms of being a cardio-protectant [24]. However, no experience has been reported with the use of liposomal formulations in addition to ACE-I or bBs, and the cumulative protective role of combined strategies is largely unknown. 

##### Clinical Scenario 2: Symptomatic Patients with a Significant Baseline Reduction in the LVEF

**Figure 2:**
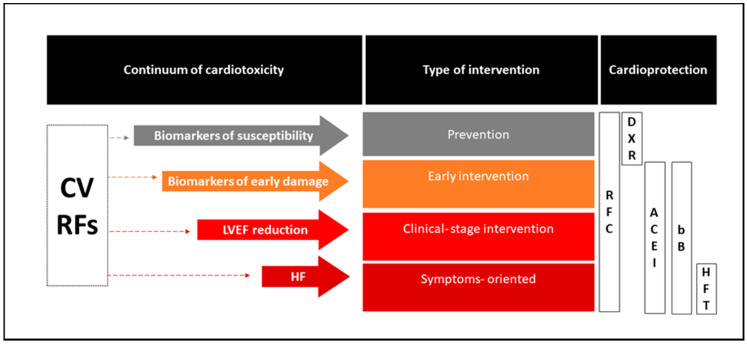
Overview of the current clinical use of cardiac protective agents across the continuum of cardiotoxicity. ACE-Is: angiotensin-converting enzyme inhibitors, bBs: beta blockers, CV: cardiovascular, DXR: dexrazoxane, HF: heart failure, HFT: heart-failure-specific therapy, LVEF: left ventricular ejection fraction, RFC: risk factors control, RFs: risk factors.

These patients meet the conditions for heart failure based on the international definitions [38,39]. All patients experiencing signs and symptoms of impaired cardiac function as a consequence of chemotherapy are patients with heart failure, and all the lessons learned from this clinical syndrome, mainly characterized for the ischemic etiology, are to be punctually considered in cancer patients. This is essential, as early recognition and prompt intervention to control heart failure can have prognostic implications [24]. If a symptomatic patient presents a baseline reduction of the LVEF between 40 and 50%, a cardiological consultation should be encouraged to produce a shared clinical strategy for treatment. Multidisciplinarity is critical for reaching a patient-centered and evidence-informed clinical decision, ensuring all the major prognostic information is well integrated and the highest standards for treatments are attained [24]. The clinical decision can be much more challenging when the baseline LVEF is <40%. In such a situation, any use of anthracycline should be discouraged, and enhanced treatment of the heart failure should be prioritized, as well as exploring alternative cancer treatments with no relevant cardiovascular toxicity. Although the majority of the experiences with chemotherapy and cardiotoxicity have focused on the anthracycline paradigm of pathogenesis, other conventional cytotoxic agents have been associated with myocardial dysfunction, including high doses of cyclophosphamide (e.g., bone-marrow transplantation setting), cisplatin, ifosfamide, and taxanes [40]; for some of them, such as cisplatin, the need to dilute the drug in high volumes of saline solution or dextran and the need for hyper-hydration to reduce the kidney toxicity can precipitate a subclinical or known cardiac dysfunction, which causes an increase of the preload and/or a secondary load of renal toxicity. When the direct toxicity of cardiomyocytes is suspected from any chemotherapy agents, the indications for the management of anthracycline-related cardiotoxicity should be extrapolated; a multidisciplinary discussion should take place to rule out the most common causes of cardiac function impairments (Figure 2). 

#### 3.2.2. Fluoropyrimidine-Induced Cardiotoxicity: Targeting Vascular Coronary Artery Toxicity

Fluoropyrimidine-related cardiotoxicity is the second most common finding after anthracycline-related damage [40]. The intravenous drug 5-fluorouracil (5-FU) or the oral capecitabine, tegafur, and trifluridine-typiracil are approved for use in a wide range of tumors, where the knowledge of the possible toxicity and their mechanisms is critical for formulating a risk-adjusted strategy for patients’ optimal management. The cardiotoxic effects of fluoropyrimidines seem to be vascular and exerted via a direct endothelial toxic activity that clinically presents as vasospasms of coronary arteries. It has been proposed that 5-FU inhibits nitric oxide (NO) synthase activity, increasing the responsiveness to vasoconstrictive stimuli with an increase of the pro-constrictive mediator endothelin-1 [41]. In the literature, the occurrence of clinically relevant cardiotoxic complications related to fluoropyrimidines has been observed in 1–18% of the cases, suggesting a significant presence in the clinical setting [42]. The overall time exposure of the endothelium to the chemotherapeutics has also been suggested to dictate the incidence risk, as cardiotoxicity has been reported in 3–8% of bolus 5-FU plus infusion administrations [40]. However, the concurrent administration of agents with potential endothelial toxic activity in poly-chemotherapy regimens, such as platinum compounds, can jeopardize the real noxious role of 5-FU in the determination of the events. The onset of ECG-alterations of ischemic significance, such as ST segment changes, symptoms suggestive of acute heart failure, LVEF new-onset dysfunction, and the entire spectrum of acute coronary artery syndromes during fluoropyrimidine treatment, strongly suggest the referral of patients to cardiologic care. However, some inducible, focal, and self-limiting ischemic events have been reported, probably due to vasospasms of the coronary arteries matching with Prinzmetal’s angina mechanisms [40]. 

In the spectrum of thrombotic-facilitating agents exerting cardiotoxicity via vascular mechanisms, both for myocardial and cerebrovascular ischemic events, cisplatin has been identified as a potential causative agent in 2% of patients [43]. The cumulative risk of cardiovascular artery disease in young patients receiving cisplatin has been estimated at around 8% within twenty years from the completion of treatments [44]. The risk of cardiovascular mortality is increased almost fivefold in the first year after a testicular cancer diagnosis in patients treated with cisplatin compared with surgery alone, which is ascribed to a possible pathological change of the endothelium with accelerated atherogenesis [44]. The current clinical indications suggest the use of vascular dilators, such as nitrates and calcium blockers, with an added anti-angina drug known as ranolazine if necessary, in patients experiencing chest pain or other cardiac symptoms during chemotherapy when pathological changes of the coronary arteries are ruled out. This indication is largely based on an experts’ consensus and is intended to allow for the dose density and intensity of cancer therapies to remain at the safest conditions [40].

#### 3.2.3. Cardiac Arrhythmias Related to Cytotoxic Chemotherapy Agents

The disruptions of the rhythm impulse generation, control, and conduction have been reported across a wide spectrum of clinical arrhythmias in cancer patients receiving active treatments. The detection of an affected rhythm has been reported in one-third of cases [40]. One common finding described in patients taking selected medicines, mainly small molecules, is the prolongation of the QT interval, which is a risk factor for a type of polymorphic ventricular tachycardia called torsade de pointes, a potentially fatal dysrhythmic event. While QT prolongation can be related to electrolyte disturbances, drug–drug interactions (including anti-emetics, such as ondansetron, and antibiotics, such as macrolides), and comorbidities (e.g., hypothyroidism), chemotherapy agents have been identified as causes in some cases. QT interval prolongation is common with anthracyclines and is observed after the first cycle of chemotherapy in more than 10% of patients [45]. The cardiotoxic effect of anthracyclines, mainly doxorubicin, seems to be exerted on the conduction system with damage to the specialized cardiomyocytes via oxidative damage [46]. Doxorubicin has been demonstrated to increase the response to pro-arrhythmogenic stimuli affecting the membrane potassium currents, increasing the susceptibility to long QT and ventricular arrhythmias when hypokalemia occurs, or during the use of drugs with effects on the repolarization phase, i.e., class III anti-arrhythmic drugs (e.g., dofetilide, ibutilide) or non-cardiological drugs, e.g., the antibiotic erythromycin [47]. Similarly, platinum compounds have been associated with QT prolongation, mainly oxaliplatin. This drug seems to delay the sodium channel closure, affecting the depolarization phase, thus increasing the risk for torsade de pointes. While the management of long QT in cancer patients is not formally different from non-cancer patients, and the onset of severe ventricular arrhythmias always prompts emergency access to cardiologic care, the administration of selected agents needs to be particularly careful. For instance, when patients present risk factors for QT prolongation, the multidisciplinary tumor board must balance the risks and benefits in the treatment decision-making and assess all the concomitant medications to reduce the risk of fatal events. In fact, this approach should be used in any similar condition with an increased risk of fatal arrhythmias, including Brugada syndrome and catecholaminergic polymorphic ventricular tachycardia, that can present serious concerns. When the corrected QT (QTc) surpasses the value of 500 msec during the ECG, temporary interruption of the cancer therapy is recommended and an evaluation of the causative etiologies should be prompted. When patients develop torsade de pointes, the usual care with magnesium sulfate is considered under specialistic care; if hemodynamic instability occurs, cardiac defibrillation is recommended [40].

Supraventricular (tachi-)arrhythmias can occur in the general population and can arise acutely in cancer patients while receiving treatments; overall, atrial fibrillation is the most common form [40]. Selected agents seem to be more prone to cause supraventricular arrhythmias; the use of gemcitabine, for example, seems to exert such an event in 8% of all the patients exposed [48]. The management of an atrial fibrillation/flutter aligns with the indications of all patients with atrial fibrillation or atrial flutter in non-cancer settings. The priorities are to control the rhythm and/or the heart rate when the atrio-ventricular response is high and reduce the risk of embolic ischemic events [40]. The choice to pursue a strategy of rate or rhythm control should be patient-centered and symptom-directed. Brady arrhythmias can also occur, for example, as a result of an increase in the parasympathetic tone or an impairment of the normal sinus node function. Paclitaxel is a plant-derived (*Taxus brevifolia*) chemotherapeutic that is associated with sinus bradycardia and is described as the single agent used for nearly one-third of the patients receiving it [49]. Though speculative, the negative chronotropic effect of paclitaxel seems to be related to the release of histamine by this drug and its solvent Cremophor EL; histamine can exert an inhibitory activity on the conductive tissue of the atrioventricular node and slow the heart rate by decreasing atrioventricular nodal conduction via histamine H1 receptors [50]. However, the common use of anti-H1 premedication agents with taxanes and the higher dilution of Cremophor nowadays has partially reduced the occurrence of this side effect of taxanes on the rhythm generation and conduction. 

### 3.3. Management of Cardiotoxicity from Non-Chemotherapy Agents: An Overview

A complete dissertation on the toxicity and management of adverse events from non-chemotherapy agents is outside the scope of the present work. Thus, bullet points have been outlined, emphasizing the most relevant effects regarding the use of these agents with chemotherapy or to remark on the main differences with chemotherapy-induced toxicities. 

#### 3.3.1. Trastuzumab and Other Anti-HER2 Agents 

Trastuzumab is an anti-HER2 monoclonal antibody that can lead to reversible dysfunction of cardiomyocytes, commonly referred to as type 2 cardiotoxicity pattern, to distinguish it from the irreversible one, which is related to anthracyclines [40]. HER2 signaling is involved in key cardiac processes for adaptive responses to stressors [51]. 

The most frequent effect of trastuzumab-related cardiac toxicity is the reduction of the LVEF. The combined analysis from phase 2 and 3 clinical trials of patients receiving trastuzumab reported an increased risk of cardiac dysfunctions (7–13%), which was amplified when combined with anthracyclines (up to 27%) [52]. The combination with a second anti-HER2 agent, such as pertuzumab or lapatinib, has not been associated with a worsened safety profile [53,54]. No excess of cardiovascular toxicity has been reported with the new antibody–drug conjugates trastuzumab emtansine (T-DM1) and trastuzumab deruxtecan (T-DXd) [55]. The cardiotoxicity from anti-HER2 agents is more pronounced in the metastatic setting and later lines of treatment, which is possibly related to the previous exposure to anthracyclines. One clinical study has been recently presented, which was designed to determine the role of ACE-I and bB in preventing trastuzumab-induced cardiotoxicity (MANTICORE study (Multidisciplinary Approach to Novel Therapies In Cardio-Oncology Research) *n* = 468 women with HER2-positive breast cancer) [56]. However, the use of lisinopril or carvedilol in this clinical trial only provided some benefits for patients exposed to trastuzumab plus anthracyclines based on the findings from a non-preplanned subgroup analysis. Accordingly, the use of these agents for the type 2 cardiotoxicity is largely investigational. 

#### 3.3.2. Immune-Related Cardiovascular Adverse Events

Immunotherapy has been rarely associated with immune-related cardiotoxic events, such as cardiomyopathy, arrhythmia, hypotension, left ventricular dysfunction, and vasculitis. The outcomes of these events can be potentially fatal. Immune-mediated iatrogenic myocarditis has received most of the attention since this results in significant morbidity and disability, and is associated with high mortality [57]. The incidence of myocarditis ranges between 0.0038 and 1.14%, as found in the pharmacovigilance registries and a retrospective analysis of the literature, respectively [58]. The management of these events is based on the use of immunosuppressors, and no role has been reported for the classic cardioprotective agents used for chemotherapy [59,60,61,62,63].

#### 3.3.3. Tyrosine Kinase Inhibitors (TKIs) and Other Small Molecules

The cardiotoxic potential of the other class of antineoplastic molecules is often undermined and underreported. A meta-analysis of more than 10,000 patients from 36 phase II and III trials showed a significantly increased risk of heart failure in people treated with small anti-VEGF molecules [64]. Some classes of drugs, for example, the MEK inhibitors used in melanoma, can exert multiple effects on cardiac function. Cobimetinib is a MEK inhibitor that can cause hypertension, LVEF reduction, and QT prolongation [65]. Such an effect has also been reported for the anti-myeloma drugs bortezomib and carfilzomib, both of which target the proteasome [66] and the multitargeted tyrosine kinase blockers vandetanib and cabozantinib. ALK inhibitors have been associated with bradycardia, especially alectinib and crizotinib. [67,68] A special caveat for the arrhythmogenic molecules needs to be mentioned regarding the Bruton’s tyrosine kinase (BTK) inhibitors [69]. Ibrutinib is an irreversible BTK blocker that is approved for the treatment of B-cell neoplasms. While it is well tolerated overall, ibrutinib has been associated with a substantially increased risk of atrial fibrillation (11.2% of patients), which requires special ECG monitoring [69,70]. 

TKI-related cardiomyopathy was also described [71]. The use of dasatinib has been associated with pulmonary hypertension, with an incidence estimated at around 3%; when not corrected, pulmonary hypertension can evolve into chronic cor pulmonale with right heart failure [72]. On the other hand, the use of nilotinib for chronic myelogenous leukemia has been associated with peripheral arterial disease in more than 6% of the patients under treatment [73]. Ischemic heart disease, ischemic cerebrovascular disease, and peripheral artery disease have also been reported, suggesting a possible toxic effect on the endothelium of arteries. 

## 4. Post-Treatment Care for Long-Term Cardiotoxic Sequelae: Survivorship Care

Survivorship care comprises a wide range of health interventions, generally as tertiary prevention. While traditionally organized as a strategy for the early detection of the disease relapse within a follow-up timeline of 5–10 years, survivorship care is now conceived as a broad health context for the holistic care of cancer survivors. Special care of long-term sequelae from cancer treatments and their implications on the prognosis is emphasized. In fact, the time of onset of heart failure from previous exposure to cardiotoxic agents can be as long as 10 years after treatments. A risk-based model of follow-up can be created for cancer survivors regarding their increased risk of experiencing adverse heart effects [8]. Such a risk stratification model of survivorship care is intended to address the important questions of priorities in the intensity of follow-up plans and provide the most appropriate care setting, in PHS and/or the specialistic setting [8]. Framing a multidisciplinary and integrated strategy for survivorship across the continuum of care as “eritis insuperabiles, si fueritis inseparabiles” (you would be insuperable if you were inseparable) can be a functional disposition for patients accessing the cardio-oncology service in a risk-stratified manner. PHS should be positioned at the center to coordinate the territorial services, which are engaged in a case-by-case manner and delivered in the primary healthcare setting and, referred to the specialistic care when appropriate. While being well-established for children, for adult cancer patients, this organizational approach has not been greatly emphasized. This warrants more implementation research to assess the safety and effectiveness of delivering cardiological pharmacotherapies for cancer patients within alternative health service delivery schemes, assuring better cost-effectiveness and resource utilization while serving patients more closely to enhance the treatment adherence and ultimately improve the outcome through a health system approach. 

## 5. Areas of Implementation and Future Perspectives

**Figure 3:**
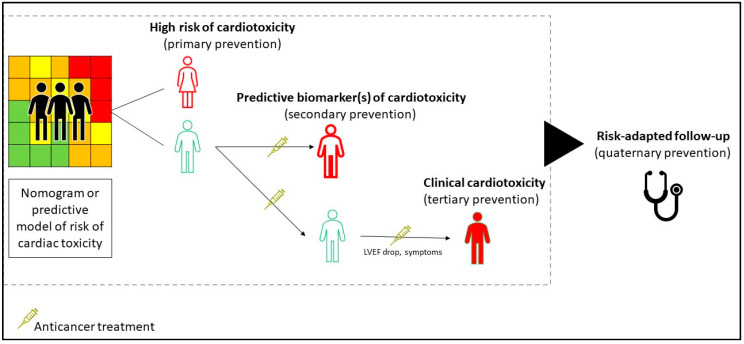
Proposed framework to identify the critical points for medical interventions to prevent and treat cardiotoxicity. The identification of cancer patients that are more susceptible to cardiac toxicity can prompt the development of interventions of primary prevention, which is adjusted for cancer types, previous treatments, and planned therapies. Validating prognostic and predictive biomarkers is critical for defining the benefits of early interventions, based on quality endpoints of cardiovascular mortality. The current strategy of the management of cardio-toxicity commonly takes the form of a tertiary type, treating patients with functional-structural alterations and cardiovascular symptoms. Eventually, the risk-adapted plans of follow-up can reduce the excessive medicalization and medical harms.

The broader context of research implementation for heart disease should now include cancer patients that are exposed to a higher risk of cardiovascular sequelae or carrying comorbidities related to the exposure to cardiotoxic antineoplastic agents. Cancer patients are traditionally under- represented in the clinical trials for cardiovascular interventions, especially for the acute coronary or arrhythmic events; this has been related to the concept that patients with cancer have indiscriminately a poor prognosis. There is no reason to separate the cardiology achievements for non-cancer patients from the cardio-oncology research field. The current drug development for cardiotoxicity mainly addresses anthracycline-related damage, either as strategies of primary prevention or the management of patients experiencing earlier signs of cardiac injury based on imaging or biomarkers (e.g., troponin) (Table 2). Though anthracycline-related damage is the most common clinical model used to implement interventions for cardiac protection, a call for innovation must be stated for the development of newer antineoplastic agents and non-anthracycline chemotherapeutics, including immunotherapy agents, and for other antineoplastic treatments. Increased research into radiation-induced heart damage is also required (Figure 3).

In the complex landscape of cancer drug development, preclinical and clinical considerations for heart damage are priorities for research and clinical care. This provides the imperative to commit to the design of patient-centered studies across the continuum of care. When committing to cancer research from our hearts, we need to take care more of patients’ hearts. 

## Figures and Tables

**Table 1:** Synoptic table of the principal studies on cardiotoxicity: prevention and management of cardiac toxicity occurring during cancer treatments.

Population (*n*)	Study Type	Cardioprotective Intervention	Outcome Measured	Benefit of the Intervention	Reference
Patients with a solid tumor or leukemia receiving Ant (*n* = 1619)	Meta-analysis	Dexrazoxane	Heart failure (clinical and subclinical)	Statistically significant benefit in favor of dexrazoxane for the occurrence of heart failure (RR: 0.29, 95% CI: 0.20–0.41, *p* < 0.00001).	Van Dalen et al., Cochrane Database Syst Rev 2011 [21]
Pediatric patients receiving Ant for AML (*n* = 1014)	Prospective, observational	Dexrazoxane	LVSD using TTE (defined as SF < 28% or EF < 55%)	Smaller EF and SF declines with dexrazoxane compared to unexposed patients across courses and a lower risk for LVSD (26.5% vs. 42.2%; HR: 0.55; 95% CI: 0.36–0.86; *p* = 0.009).	Getz et al., J. Clin. Oncol. 2020 [32]
BC patients receiving Ant (*n* = 83)	Prospective, randomized, placebo-controlled, double-blind	Spironolactone	LVEF using TTE	LVEF decreased from 67.0 ± 6.1% to 65.7 ± 7.4% (*p* = 0.094) in the spironolactone group, and from 67.7 ± 6.3% to 53.6 ± 6.8% in the control group (*p* < 0.001).	Akpek et al., Eur J Heart Fail 2015 [27]
Early BC patients receiving adjuvant Ant +/- trastuzumab and RT (*n* = 120)	2 × 2 factorial, randomized, placebo-controlled, double-blind	Candesartan, metoprolol, or matching placebos	LVEF using cardiac MRI	The overall decline in LVEF was 2.6% (95% CI: 1.5–3.8%) in the placebo group and 0.8% (95% CI: 0.4–1.9%) in the candesartan group in the intention-to-treat analysis (*p* = 0.026). No effect of metoprolol on the overall decline in LVEF.	Gulati et al., Eur Heart J 2016 [28]
Patients with malignant memopathies that were eligible for intensive chemotherapy (*n* = 90)	Randomized, controlled	Enalapril and carvedilol	LVEF using TTE and MRI	LVEF did not change in the intervention group but significantly decreased in the controls, resulting in a −3.1% absolute difference based on echocardiography (*p* = 0.035) and −3.4% (*p* = 0.09) in the 59 patients who underwent MRI.	Bosch et al., Jam Coll Cardiol 2013 [29]
Patients with HER2-negative early BC receiving Ant (*n* = 200)	Prospective, randomized, placebo-controlled, double-blind	Carvedilol	Early-onset decrease in LVEF ≥ 10% at 6 months	Primary endpoint occurred in 14 patients (14.5%) in the carvedilol group and 13 patients (13.5%) in the placebo group (*p* = 1.0). No differences in changes in LVEF were noted between groups.	Avila et al., J Am Coll Cardiol. 2018 [30]
Patients with advanced BC treated with Ant (*n* = 120)	Prospective, blinded, observational	Digitalo-diuretic therapy + ACE inhibitor	Recovery of the LVEF after developing CHF	Cardiac function continued to deteriorate during digitalo-diuretic therapy for 3 months. Almost all LVEF values returned to normal after a median of 3 months of ACE inhibitor therapy and remained stable in the follow-up period (median of 33 months).	Jensen et al. Ann Oncol. 2002 [33]
Long-term survivors of pediatric cancer exposed to Ant, with a cardiac abnormality identified (*n* = 135)	Randomized, double-blind, controlled	Enalapril	Cardiac function deterioration (defined using MCI on an exercise test or an increase in LVESWS)	No difference in the rate of change in MCI between enalapril and the placebo groups. The rate of change in LVESWS was greater in the enalapril group than in the placebo group (−8.59 vs. 1.85 g/cm [2]; *p* = 0.033) during the first year and maintained over time, resulting in a 9% reduction in the estimated LVESWS by year 5 in the enalapril group.	Silber et al., J. Clin. Oncol. 2004 [34]
Patients with hematologic or solid tumors with LVEF ≤ 45% due to AC-CMP (*n* = 201)	Prospective study	Enalapril and, when possible, carvedilol	Recovery of the LVEF using TTE	42% of patients were responders (LVEF ≥ 50%), 13% were partial responders (10% < LVEF ≤50%), and 45% were non-responders (LVEF increase ≤ 10%). Responders showed a lower rate of cumulative cardiac events than partial and non-responders (5%, 31%, and 29%, respectively; *p* < 0.001).	Cardinale et al., J Am Coll Cardiol. 2010 [35]
Adult patients treated with Ant (*n* = 273)	Controlled, open-label, phase III	Enalapril started before Ant (prevention arm) or at troponin increase (troponin-triggered arm)	Incidence of troponin elevation	No difference in the proportion of patients with a first high troponin level: 23% in the prevention group and 26% in the troponin-triggered group (*p* = 0.50), or in the time to the first troponin elevation (HR: 1.13, 95% CI: 0.70–1.83; *p* = 0.61). The median level of the first elevation of troponin was 40% (22–90%) above the ULN in the prevention group and 33% (18–50%) in the troponin-triggered arm (*p* = 0.17).	Cardinale et al., Eur J Cancer. 2018 [36]

AC-CMP: anthracycline-induced cardiomyopathy, ACE: angiotensin-converting enzyme, Ant: anthracycline, AML: acute myeloid leukemia, BC: breast cancer, CHF: congestive heart failure, EF: ejection fraction, HR: hazard ratio, LVEF: left ventricular ejection fraction, LVESWS: left ventricular end-systolic wall stress, LVSD: left ventricular systolic dysfunction, MCI: maximal cardiac index, MRI: magnetic resonance imaging, RT: radiation therapy, SF: shortening fraction, TTE: transthoracic echocardiography, ULN: upper limit of normality.

**Table 2:** An overview of the ongoing clinical trials of cardio-protective agents with patients receiving anticancer drugs.

Population	Intervention	Pharmacological Class	Phase	Primary Outcome	NCT Identifier
Patients with ES, OS, and AML scheduled for chemotherapy	Captopril	ACE-I	3	Effect of ACE-Is in preventing chemotherapy-related cardiotoxicity	NCT03389724
Patients scheduled for anthracycline	Ivabradine	Selective inhibitor of If	NA	Reduction in the global longitudinal strain of at least 10%	NCT03650205
Early breast cancer patients eligible for anthracycline +/- trastuzumab	Bisoprolol; ramipril	bB and ACE-I	3	Maximum change in the LVEF	NCT02236806 (SAFE)
Breast cancer patients eligible for anthracycline treatment	Sulforaphane	Nutritional supplement	1/2	Change in cardiac function after doxorubicin	NCT03934905
NHL patients scheduled for (R)CHOP type treatments	Atorvastatin	Lipid- lowering statin	2	LVEF preservation at 12 months	NCT02943590 (STOP-CA)
Adolescent patients after a bone marrow transplantation for hematological malignancies	Sacubitril, valsartan	Neprilysin inhibitor and ACE-I	NA	Change in the left ventricular function	NCT04092309
Breast cancer patients treated with doxorubicin	Alfacalcidol	Vitamin D	2	Change in the plasma levels of troponin-T	NCT04166253
Breast cancer patients scheduled for anthracycline	Alpha-lipoic acid	Dietary supplement	NA	Serum brain natriuretic peptide, neurotensin, and TNF-α level plasma assessment	NCT03908528
Early breast cancer patients eligible for anthracycline	Xinmailong	Bioactive fraction extracted from *Periplaneta Americana* (American cockroach)	2	Rate of no cardiac events during chemotherapy	NCT03785704
Early breast cancer patients eligible for anthracycline	Atorvastatin	Lipid- lowering statin	2	LVEF preservation at 24 months	NCT01988571 (PREVENT)
Chemotherapy patients at risk of cardiotoxicity	ACE-I and bB	ACE-I and bB	NA	New LV dysfunction, as defined based on a 3D echo	ACTRN12614000341628 (SUCCOUR)

Source: NCTtrial.gov (last access: 31 August 2020). ACE-I: angiotensin-converting enzyme inhibitor; AML: acute myeloblastic leukemia; bB: beta blocker; ES: Ewing sarcoma; If: funny channel current; LV: left ventricle; LVEF: left ventricular ejection fraction; NA: not applicable; NCT: National clinical trial; OS: osteosarcoma; (R)CHOP: chemotherapy protocol with (Rituximab) cyclophosphamide, doxorubicin, vincristine, and prednisolone.

## References

[1] Siegel R.L., Miller K.D., Jemal A. (2020). Cancer statistics, 2020. CA Cancer J. Clin..

[2] Lee L., Cheung W.Y., Atkinson E., Krzyzanowska M.K. (2011). Impact of comorbidity on chemotherapy use and outcomes in solid tumors: A systematic review. J. Clin. Oncol..

[3] Lenihan D.J., Oliva S., Chow E.J., Cardinale D. (2013). Cardiac toxicity in cancer survivors. Cancer.

[4] GBD 2015 Risk Factors Collaborators (2016). Global, regional, and national comparative risk assessment of 79 behavioural, environmental and occupational, and metabolic risks or clusters of risks, 1990–2015: A systematic analysis for the Global Burden of Disease. Lancet.

[5] Oni T., Mogo E., Ahmed A., Davies J.I. (2019). Breaking down the silos of Universal Health Coverage: Towards systems for the primary prevention of non-communicable diseases in Africa. BMJ Glob. Health.

[6] World Health Organization Global Action Plan for Healthy Lives and Well-Being for All. https://www.who.int/sdg/global-action-plan/Global_Action_Plan_Phase_I.pdf.

[7] Tonorezos E.S., Conigliaro J. (2017). Integration of cancer survivorship care and primary care practice. JAMA Intern. Med..

[8] Mc Cabe M.S., Partridge A.H., Grunfeld E., Hudson M.M. (2013). Risk-based health care, the cancer survivor, the oncologist, and the primary care physician. Semin. Oncol..

[9] Yoon G.J., Telli M.L., Kao D.P., Matsuda K.Y., Carlson R.W., Witteles R.M. (2010). Left ventricular dysfunction in patients receiving cardiotoxic cancer therapies are clinicians responding optimally?. J. Am. Coll. Cardiol..

[10] WHO CVD Risk Chart Working Group (2019). World Health Organization cardiovascular disease risk charts: Revised models to estimate risk in 21 global regions. Lancet Glob. Health.

[11] Bray F., Ferlay J., Soerjomataram I., Siegel R.L., Torre L.A., Jemal A. (2018). Global cancer statistics 2018: GLOBOCAN estimates of incidence and mortality worldwide for 36 cancers in 185 countries. CA Cancer J. Clin..

[12] Lee K., Brumme Z.L. (2013). Operationalizing the One Health approach: The global governance challenges. Health Policy Plan..

[13] Parent S., Pituskin E., Paterson D.I. (2016). The Cardio-oncology Program: A Multidisciplinary Approach to the Care of Cancer Patients With Cardiovascular Disease. Can. J. Cardiol..

[14] Weiss R.B. (1992). The anthracyclines: Will we ever find a better doxorubicin?. Semin. Oncol..

[15] Jimenez Hernandez R.M., Antolín J.M.S., Hernandez R.M.J., Calle P.T., Ruigomez A.C., Arrojo S.D.C., Abad C.G., Varela C.C., Martín J.J.A. (2020). Incidence of long-term cardiotoxicity and evolution of the systolic function in patients with breast cancer treated with anthracyclines. Cardiol. J..

[16] Zeeneldin A.A., Sallam Y.A., Gaber A.A., Shaheen A.A. (2015). Non-anthracycline chemotherapy associated with a poor outcome in elderly Egyptian patients with diffuse large B-cell non-Hodgkin lymphoma. J. Cancer Metastasis Treat..

[17] McGowan J.V., Chung R., Maulik A., Piotrowska I., Walker J.M., Yellon D.M. (2017). Anthracycline Chemotherapy and Cardiotoxicity. Cardiovasc. Drugs Ther..

[18] Cardinale D., Colombo A., Bacchiani G., Tedeschi I., Meroni C.A., Veglia F., Civelli M., LaMantia G., Colombo N., Curigliano G. (2015). Early detection of anthracycline cardiotoxicity and improvement with heart failure therapy. Circulation.

[19] Jones R.L. (2008). Utility of dexrazoxane for the reduction of anthracycline-induced cardiotoxicity. Expert Rev. Cardiovasc. Ther..

[20] Reichardt P., Tabone M.D., Mora J., Morland B., Jones R.L. (2018). Risk-benefit of dexrazoxane for preventing anthracycline-related cardiotoxicity: Re-evaluating the European labeling. Future Oncol..

[21] van Dalen E.C., Caron H.N., Dickinson H.O., Kremer L.C. (2011). Cardioprotective interventions for cancer patients receiving anthracyclines. Cochrane Database Syst. Rev..

[22] Asselin B.L., Devidas M., Chen L., Franco V.I., Pullen J., Borowitz M.J., Hutchison R.E., Ravindranath Y., Armenian S.H., Camitta B.M. (2016). Cardioprotection and safety of dexrazoxane in patients treated for newly diagnosed T-cell acute Lymphoblastic leukemia or advanced-stage lymphoblastic non-Hodgkin Lymphoma: A Report of the Children’s Oncology Group Randomized Trial Pediatric Oncology Group 9404. J. Clin. Oncol..

[23] European Medicines Agency Committee for Medicinal Products for Human Use (EMA/398612/2017, 18th May 2017). Assessment Report for Dexrazoxane. https://www.ema.europa.eu/en/documents/referral/cardioxane-article-13-referral-chmp-assessment-rep..

[24] Curigliano G., Lenihan D., Fradley M., Ganatra S., Barac A., Blaes A., Herrmann J., Porter C., Lyon A., Lancellotti P. (2020). Management of cardiac disease in cancer patients throughout oncological treatment: ESMO consensus recommendations. Ann. Oncol..

[25] Oparil S., Haber E. (1974). The renin-angiotensin system. N. Engl. J. Med..

[26] Pinter M., Kwanten W.J., Jain R.K. (2018). Renin-Angiotensin System Inhibitors to Mitigate Cancer Treatment-Related Adverse Events. Clin. Cancer Res..

[27] Akpek M., Ozdogru I., Sahin O., Inanc M., Dogan A., Yazici C., Berk V., Karaca H., Kalay N., Oguzhan A. (2015). Protective effects of spironolactone against anthracycline-induced cardiomyopathy. Eur. J. Heart Fail..

[28] Gulati G., Heck S.L., Ree A.H., Hoffmann P., Schulz-Menger J., Fagerland M.W., Gravdehaug B., Von Knobelsdorff-Brenkenhoff F., Bratland Å., Storås T.H. (2016). Prevention of cardiac dysfunction during adjuvant breast cancer therapy (PRADA): A 2 × 2 factorial, randomized, placebo-controlled, double-blind clinical trial of candesartan and metoprolol. Eur. Heart J..

[29] Bosch X., Rovira M., Sitges M., Domènech A., Ortiz-Pérez J.T., De Caralt T.M., Morales-Ruiz M., Perea R.J., Monzo M., Esteve J. (2013). Enalapril and carvedilol for preventing chemotherapy-induced left ventricular systolic dysfunction in patients with malignant hemopathies: The OVERCOME trial (preventiOn of left Ventricular dysfunction with Enalapril and caRvedilol in patients submitted to intensive ChemOtherapy for the treatment of Malignant hEmopathies). J. Am. Coll. Cardiol..

[30] Avila M.S., Ayub-Ferreira S.M., de Barros Wanderley M.R., das Dores Cruz F., Gonçalves Brandão S.M., Rigaud V.O.C., Higuchi-Dos-Santos M.H., Hajjar L.A., Filho R.K., Hoff P.M. (2018). Carvedilol for Prevention of Chemotherapy-Related Cardiotoxicity: The CECCY Trial. J. Am. Coll. Cardiol..

[31] Chow S.L., Maisel A.S., Anand I., Bozkurt B., de Boer R.A., Felker G.M., Fonarow G.C., Greenberg B., Januzzi J.L., Kiernan M.S. (2017). Role of Biomarkers for the Prevention, Assessment, and Management of Heart Failure: A Scientific Statement From the American Heart Association. Circulation.

[32] Getz K.D., Sung L., Alonzo T.A., Leger K.J., Gerbing R.B., Pollard J.A., Cooper T., Kolb E.A., Gamis A.S., Ky B. (2020). Effect of Dexrazoxane on Left Ventricular Systolic Function and Treatment Outcomes in Patients With Acute Myeloid Leukemia: A Report From the Children’s Oncology Group. J. Clin. Oncol..

[33] Jensen B.V., Skovsgaard T., Nielsen S.L. (2002). Functional monitoring of anthracycline cardiotoxicity: A prospective, blinded, long-term observational study of outcome in 120 patients. Ann. Oncol..

[34] Silber J.H., Cnaan A., Clark B.J., Paridon S.M., Chin A.J., Rychik J., Hogarty A.N., Cohen M.I., Barber G., Rutkowski M. (2004). Enalapril to prevent cardiac function decline in long-term survivors of pediatric cancer exposed to anthracyclines. J. Clin. Oncol..

[35] Cardinale D., Colombo A., Lamantia G., Colombo N., Civelli M., De Giacomi G., Rubino M., Veglia F., Fiorentini C., Cipolla C.M. (2010). Anthracycline-induced cardiomyopathy: Clinical relevance and response to pharmacologic therapy. J. Am. Coll. Cardiol..

[36] Cardinale D., Ciceri F., Latini R., Franzosi M.G., Sandri M.T., Civelli M., Cucchi G., Menatti E., Mangiavacchi M., Cavina R. (2018). Anthracycline-induced cardiotoxicity: A multicenter randomised trial comparing two strategies for guiding prevention with enalapril: The International CardioOncology Society-one trial. Eur. J. Cancer.

[37] Meessen J.M.T.A., Cardinale D., Ciceri F., Sandri M.T., Civelli M., Bottazzi B., Cucchi G., Menatti E., Mangiavacchi M., Condorelli G. (2020). Circulating biomarkers and cardiac function over 3 years after chemotherapy with anthracyclines: The ICOS-ONE trial. ESC Heart Fail..

[38] Ponikowski P., Voors A.A., Anker S.D., Bueno H., Cleland J.G.F., Coats A.J.S., Falk V., González-Juanatey J.R., Harjola V.-P., Jankowska E.A. (2016). 2016 ESC Guidelines for the diagnosis and treatment of acute and chronic heart failure: The Task Force for the diagnosis and treatment of acute and chronic heart failure of the European Society of Cardiology (ESC)Developed with the special contribution of the Heart Failure Association (HFA) of the ESC [published correction appears in Eur Heart J. 2016 Dec 30]. Eur. Heart J..

[39] Yancy C.W., Jessup M., Bozkurt B., Butler J., Casey D.E., Colvin M.M., Drazner M.H., Filippatos G.S., Fonarow G.C., Givertz M.M. (2017). ACC/AHA/HFSA Focused Update of the 2013 ACCF/AHA Guideline for the Management of Heart Failure: A Report of the American College of Cardiology/American Heart Association Task Force on Clinical Practice Guidelines and the Heart Failure Society of America. Circulation.

[40] Zamorano J.L., Lancellotti P., Rodriguez Muñoz D., Aboyans V., Asteggiano R., Galderisi M., Habib G., Lenihan D.J., Lip G.Y.H., Lyon A.R. (2016). ESC Position Paper on cancer treatments and cardiovascular toxicity developed under the auspices of the ESC Committee for Practice Guidelines: The Task Force for cancer treatments and cardiovascular toxicity of the European Society of Cardiology (ESC). Eur. Heart J..

[41] Lamberti M., Porto S., Zappavigna S., Addeo E., Marra M., Miraglia N., Sannolo N., Vanacore D., Stiuso P., Caraglia M. (2014). A mechanistic study on the cardiotoxicity of 5-fluorouracil in vitro and clinical and occupational perspectives. Toxicol. Lett..

[42] Van Cutsem E., Hoff P.M., Blum J.L., Abt M., Osterwalder B. (2002). Incidence of cardiotoxicity with the oral fluoropyrimidine capecitabine is typical of that reported with 5-fluorouracil. Ann. Oncol..

[43] Moore R.A., Adel N., Riedel E., Bhutani M., Feldman D.R., Tabbara N.E., Soff G.A., Parameswaran R., Hassoun H. (2011). High incidence of thromboembolic events in patients treated with cisplatin-based chemotherapy: A large retrospective analysis. J. Clin. Oncol..

[44] Cameron A.C., Touyz R.M., Lang N.N. (2016). Vascular Complications of Cancer Chemotherapy. Can. J. Cardiol..

[45] Horacek J.M., Jakl M., Horackova J., Pudil R., Jebavy L., Maly J. (2009). Assessment of anthracycline-induced cardiotoxicity with electrocardiography. Exp. Oncol..

[46] Simůnek T., Stérba M., Popelová O., Adamcová M., Hrdina R., Gersl V. (2009). Anthracycline-induced cardiotoxicity: Overview of studies examining the roles of oxidative stress and free cellular iron. Pharmacol. Rep..

[47] Milberg P., Fleischer D., Stypmann J., Osada N., Mönnig G., Engelen M.A., Bruch C., Breithardt G., Haverkamp W., Eckardt L. (2007). Reduced repolarization reserve due to anthracycline therapy facilitates torsade de pointes induced by IKr blockers. Basic Res. Cardiol..

[48] Gridelli C., Cigolari S., Gallo C., Manzione L., Ianniello G.P., Frontini L., Ferraù F., Robbiati S.F., Adamo V., Gasparini G. (2001). Activity and toxicity of gemcitabine and gemcitabine + vinorelbine in advanced non-small-cell lung cancer elderly patients: Phase II data from the Multicenter Italian Lung Cancer in the Elderly Study (MILES) randomized trial. Lung Cancer.

[49] McGuire W.P., Rowinsky E.K., Rosenshein N.B., Grumbine F.C., Ettinger D.S., Armstrong D.K., Donehower R.C. (1989). Taxol: A unique antineoplastic agent with significant activity in advanced ovarian epithelial neoplasms. Ann. Intern. Med..

[50] Nault M.A., Milne B., Parlow J.L. (2002). Effects of the Selective H1 and H2Histamine Receptor Antagonists Loratadine and Ranitidine on Autonomic Control of the Heart. Anesthesiology.

[51] D’Uva G., Aharonov A., Lauriola M., Kain D., Yahalom-Ronen Y., Carvalho S., Weisinger K., Bassat E., Rajchman D., Yifa O. (2015). ERBB2 triggers mammalian heart regeneration by promoting cardiomyocyte dedifferentiation and proliferation. Nat. Cell Biol..

[52] Seidman A., Hudis C., Pierri M.K., Shak S., Paton V., Ashby M., Murphy M., Stewart S.J., Keefe D. (2002). Cardiac dysfunction in the trastuzumab clinical trials experience. J. Clin. Oncol..

[53] Perez E.A., Koehler M., Byrne J., Preston A.J., Rappold E., Ewer M.S. (2008). Cardiac safety of lapatinib: Pooled analysis of 3689 patients enrolled in clinical trials. Mayo Clin. Proc..

[54] Swain S.M., Ewer M.S., Cortés J., Amadori D., Miles D., Knott A., Clark E., Benyunes M.C., Ross G., Baselga J. (2013). Cardiac tolerability of pertuzumab plus trastuzumab plus docetaxel in patients with HER2-positive metastatic breast cancer in CLEOPATRA: A randomized, double-blind, placebo-controlled phase III study. Oncologist.

[55] Pondé N., Ameye L., Lambertini M., Paesmans M., Piccart M., De Azambuja E. (2020). Trastuzumab emtansine (T-DM1)-associated cardiotoxicity: Pooled analysis in advanced HER2-positive breast cancer. Eur. J. Cancer.

[56] Guglin M., Krischer J., Tamura R., Fink A., Bello-Matricaria L., McCaskill-Stevens W., Munster P.N. (2019). Randomized Trial of Lisinopril Versus Carvedilol to Prevent Trastuzumab Cardiotoxicity in Patients With Breast Cancer. J. Am. Coll. Cardiol..

[57] Puzanov I., Diab A., Abdallah K., Bingham C.O., Brogdon C., Dadu R., Hamad L., Kim S., Lacouture M.E., LeBoeuf N.R. (2017). Managing toxicities associated with immune checkpoint inhibitors: Consensus recommendations from the Society for Immunotherapy of Cancer (SITC) Toxicity Management Working Group. J. Immunother. Cancer.

[58] Salem J.E., Manouchehri A., Moey M., Lebrun-Vignes B., Bastarache L., Pariente A., Gobert A., Spano J.-P., Balko J.M., Bonaca M.P. (2018). Cardiovascular toxicities associated with immune checkpoint inhibitors: An observationalretrospective, pharmacovigilance study. Lancet Oncol..

[59] Mahmood S.S., Fradley M.G., Cohen J.V., Nohria A., Reynolds K.L., Heinzerling L.M., Sullivan R.J., Damrongwatanasuk R., Chen C.L., Gupta D. (2018). Myocarditis in patients treated with immune checkpoint inhibitors. J. Am. Coll. Cardiol..

[60] Heinzerling L., Ott P.A., Hodi F.S., Husain A.N., Tajmir-Riahi A., Tawbi H., Pauschinger M., Gajewski T.F., Lipson E.J., Luke J.J. (2016). Cardiotoxicity associated with CTLA4 and PD1 blocking immunotherapy. J. Immunother. Cancer.

[61] Santos R.C., Figueiredo V.N., Martins L.C., de Haro Moraes C., Quinaglia T., Boer-Martins L., Ferreira-Melo S.E., Yazbek M.A., Bertolo M. (2012). Heitor Moreno Infliximab reduces cardiac output in rheumatoid arthritis patients without heart failure. Rev. Assoc. Med. Bras..

[62] Esfahani K., Buhlaiga N., Thébault P., Lapointe R., Johnson N.A., Miller W.H. (2019). Alemtuzumab for Immune-Related Myocarditis Due to PD-1 Therapy. N. Engl. J. Med..

[63] Varricchi G., Galdiero M.R., Marone G., Criscuolo G., Triassi M., Bonaduce D., Marone G., Tocchetti C.G. (2017). Cardiotoxicity of immune checkpoint inhibitors. ESMO Open.

[64] Qi W.X., Shen Z., Tang L.N., Yao Y. (2014). Congestive heart failure risk in cancer patients treated with vascular endothelial growth factor tyrosine kinase inhibitors: A systematic review and meta-analysis of 36 clinical trials. Br. J. Clin. Pharmacol..

[65] Mincu R.I., Mahabadi A.A., Michel L., Mrotzek S.M., Schadendorf D., Rassaf T., Totzeck M. (2019). Cardiovascular adverse events associated with BRAF and MEK inhibitors: A systematic review and meta-analysis. JAMA Netw. Open.

[66] Siegel D., Martin T., Nooka A., Harvey R.D., Vij R., Niesvizky R., Badros A.Z., Jagannath S., McCulloch L., Rajangam K. (2013). Integrated safety profile of single-agent carfilzomib: Experience from 526 patients enrolled in 4 phase II clinical studies. Haematologica.

[67] Ou S.H., Tang Y., Polli A., Wilner K.D., Schnell P. (2016). Factors associated with sinus bradycardia during crizotinib treatment: A retrospective analysis of two large-scale multinational trials (PROFILE 1005 and 1007). Cancer Med..

[68] Ghatalia P., Je Y., Kaymakcalan M.D. (2015). QTc interval prolongation with vascular endothelial growth factor receptor tyrosine kinase inhibitors. Br. J. Cancer.

[69] Leong D.P., Caron F., Hillis C., Duan A., Healey J.S., Fraser G., Siegal D.M. (2016). The risk of atrial fibrillation with ibrutinib use: A systematic review and meta-analysis. Blood.

[70] Shanafelt T.D., Parikh S.A., Noseworthy P.A., Goede V., Chaffee K.G., Bahlo J., Call T.G., Schwager S.M., Ding W., Eichhorst B. (2017). Atrial fibrillation in patients with chronic lymphocytic leukemia (CLL). Leuk Lymphoma.

[71] Moslehi J.J., Deininger M. (2015). Tyrosine Kinase Inhibitor-Associated Cardiovascular Toxicity in Chronic Myeloid Leukemia. J. Clin. Oncol..

[72] Jabbour E., Kantarjian H.M., Saglio G., Steegmann J.L., Shah N.P., Boqué C.J., Chuah C., Pavlovsky C., Mayer J., Cortes J. (2014). Early response with dasatinib or imatinib in chronic myeloid leukemia: 3-year follow-up from a randomized phase 3 trial (DASISION). Blood.

[73] Le Coutre P., Rea D., Abruzzese E., Dombret H., Trawinska M.M., Herndlhofer S., Dörken B., Valent P. (2011). Severe peripheral arterial disease during nilotinib therapy. J. Natl. Cancer Inst..

